# Mycotoxins in Tea: Occurrence, Methods of Determination and Risk Evaluation

**DOI:** 10.3390/toxins10110444

**Published:** 2018-10-30

**Authors:** Irina Sedova, Mariya Kiseleva, Victor Tutelyan

**Affiliations:** Federal Research Centre of Nutrition, Biotechnology and Food Safety, Ust’inskiy pr., 2/14, Moscow 109240, Russia; mg_kiseleva@ion.ru (M.K.); tutelyan@ion.ru (V.T.)

**Keywords:** tea, Pu-erh, *Camellia sinensis*, moulds, mycotoxins, occurrence, food safety, exposure, methods of determination

## Abstract

Tea is one of the most popular beverages all over the world. Being an everyday drink for almost everyone, for centuries tea was considered safe and healthy. However, fungal contamination of tea at any stage of commodity production can pose a serious health hazard due to the accumulation of toxic secondary metabolites of moulds. Contemporary research revealed incidences of highly contaminated samples. Mycotoxin transfer from naturally contaminated raw tea into beverage was well studied for ochratoxin A only, and the possible leak of other mycotoxins is discussed. The results of several surveys were combined to evaluate aflatoxin B1 and ochratoxin A contamination levels in black tea and Pu-erh. Exposure estimate to aflatoxin B1 and ochratoxin A due to tea consumption was carried out based on these data. Average contamination level corresponds to the exposure of 3–40% (aflatoxin B1) and 5–24% (ochratoxin A) of mean overall estimates for different cluster diets. Lack of data does not allow the conclusion for the necessity of public health protection measures. It is necessary to perform representative studies of different kinds of tea for regulated mycotoxins at least. Contemporary techniques for analysis of mycotoxins in tea are summarised in the present review.

## 1. Introduction

Tea is an aromatic beverage commonly prepared by pouring hot or boiling water over cured leaves of the *Camellia sinensis*, an evergreen bush native to East Asia [1]. It is an everyday drink for almost everyone. A statistical study carried out in 2017 in the USA, the UK, and Germany showed 30% to 40% of respondents drink two to three cups of tea per day [2]. Tea is generally divided into categories based on fermentation degree. The most familiar kinds are black, white, oolong, green, and Pu-erh (post-fermented) tea. There are also teas flavored by the addition of mint, vanilla etc. and herbal teas, consisting of fruits and herbs, not *Camellia sinensis*. China, India, Kenya, Sri Lanka, and Turkey are the world’s largest tea producers. China and India accounted for about 43% and 22% of world tea production, respectively. According to the Food and Agriculture Organization of the United Nations Intergovernmental Group on tea, its consumption has increased by 4.5% annually over the last decade. By 2027, the world black tea production is projected to increase by an annual growth rate of 2.2% and green tea by 7.5% [3].

Consumption growth, especially of green tea, is believed to be accounted for by healthy lifestyle trends. Health benefits are associated with vitamins, microelements, essential oils, and polyphenols [4]. Two significant groups of tea polyphenols are catechins and flavonols. Catechins are abundant in less-fermented tea; epigallocatechin gallate may account for 50–80% of the total catechin (75–150 mg in a typical tea beverage) in tea. Flavonols are quercetin, kaempferol, myricetin, and their glycosides [5]. Tea polyphenols possess the bioactivity to affect the pathogenesis of some chronic diseases due to their antioxidant, anti-inflammatory, antiproliferative, antimutagenic, antibacterial and antiviral properties, protection against cardiovascular disease, hyperglycaemia, metabolic disorders, and some cancers [6,7]. However, a positive impact on health may be devalued by the presence of harmful contaminants, such as heavy metals, mycotoxins, and pesticide residues [6,8].

Mycotoxins are abiotic hazards produced by certain fungi that can grow on a variety of crops [9]. Mycotoxin production in tea can occur at any stage of its manufacturing: tea bush cultivation, harvest, processing, and storage. Poor agricultural practices, improper processing, drying, packaging, storage, and transport conditions promote fungal growth, increasing the risk of mycotoxin contamination. A subtropical climate, being favourable for tea cultivation, is also suitable for toxinogenic mould growth. Aflatoxins and ochratoxin A are one the most potent health hazards. Moreover, China and India—major tea producers—are in the list of the most aflatoxin affected countries [10]. It is reasonable that aflatoxins and ochratoxin A are the first candidates to be traced in tea. Most of the research concerns determination of the above mycotoxins. Their content in tea reached dozens and hundreds of ppb, respectively. Modern analytical techniques afford the development of multi-analyte and even multi-class methods. Recent issues aimed at multi-mycotoxin analyses revealed tea sample contamination with fumonisins, deoxynivalenol, and enniatins. The present study aims to summarise available data on fungi and mycotoxin occurrence in green, black, and Pu-erh tea. However, there is a distinct lack of representative and trustworthy issues to generate an accurate picture.

## 2. Moulds in Tea

**Figure 1:**
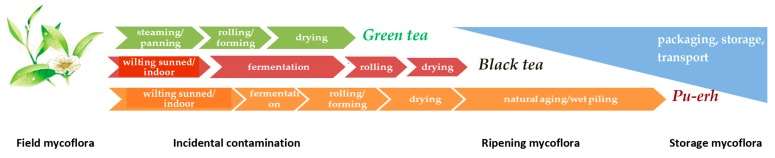
The common types of tea processing stages and associated mycoflora.

Tea fungal contamination can occur at any stage of its production. Depending on the type, tea can be subjected to various mycoflora invasion, as shown in Figure 1. A wet and warm climate favorable for tea cultivation is also suitable for fungal growth. The principal genus identified in soils from subtropical tea plantations in China is *Fusarium* [11]. “Field” mycoflora is responsible for such mycotoxin production as deoxynivalenol and its derivatives, T-2 and HT-2 toxins, fumonisins etc. Subsequent steps of tea production, storage, and delivery to consumer impact contamination also [12]. Thirty-four fungal species were isolated from air, phyllosphere, and soil samples from a tea factory, including mycotoxin producing moulds (*Aspergillus niger*, *A. flavus*, *A. fumigatus*, and *Fusarium lactis*) [13]. Green tea manufacturing is the quickest: fresh leaves from plantations are subjected to immediate steaming or panning to deactivate oxidising enzymes and retain polyphenols. Steaming is followed by rolling and drying. Thus, green tea is expected to be the least contaminated at the processing stages. Compared to green, black tea production is more time consuming: wilting and fermentation steps are added to promote enzymatic oxidation and subsequent condensation of tea polyphenols into theaflavins and thearubigins [7,14].

Mycological study of 40 black and green, loose and bagged tea samples from the Czech Republic revealed total fungal contamination [15], and similar results were obtained for 32 samples from Italy [16].

*Aspergillus*, *Penicillium*, *Rhizopus*, *Eurotium*, *Cladosporium*, and *Trichoderma* genera species were identified in tea, the first two dominating [15,16,17,18,19,20,21]. *Aspergillus* genera was usually represented by *A. niger* in black tea (Oman) [18]; black and green tea (Czech Republic) [15]; *A. niger* and *A. tubingensis* in green and black bagged tea (Italy) [16]; and *A. niger*, *A. acidus*, *A. awamori*, *A. tubingensis*, and *A. carbonarius* in herbal tea (Sweden) [22]. According to Rezacova and Kubatova, fermentation or plant origin did not affect tea fungal community, only storage mycoflora (saprobic fungi preferring dried food) was found [15]. Possible “field” mycoflora, even being not found in stored tea (approximately a year of proper storage results in mycelium loss [23]), can be traced by mycotoxins—its secondary metabolites. For example, detection of deoxynivalenol or enniatins in a tea sample is an indication of pre-harvest plant contamination by *Fusarium* species. Fresh leaf mycological examination could identify possible *Camellia sinensis* phytopathogens.

Post-fermented tea Pu-erh needs a dozen years of maturation to be considered ripened [24]. Wet piling is an alternative, affording to speed up the process. Regardless, the post-fermentation stage welcomes fungal contamination, and Pu-erh tea mycoflora attracts special attention. The common fungi isolated during the fermentation process of Pu-erh tea mainly belongs to *Aspergillus*, yeasts, *Penicillium*, *Rhizopus*, and *Mucor* [25]. The fungal and bacterial diversity of raw and ripened Pu-erh tea was well studied by Zhang [26]. The most abundant moulds found were *A. niger* (85% and 60% for 60 samples of ripe and raw Pu-erh), *A. penicilloides*, and *A. cibarius*. Ripening resulted in the occurrence of *Penicillium* species, such as *P. brocae* and *P*. *citrinum* [27]. Mogensen found *A. acidus* to be predominant in 10 samples of Pu-erh [21]. *A. acidus* and *A. fumigatus* frequency reached 50–80% in 36 Pu-erh samples studied by Haas et al., and *Rhizomucor*, *Mycelia*, *Mucor*, and *Penicillium* (*P. citrinum*, *P. commune*) fungal taxa were also detected [28].

## 3. Mycotoxins in Tea

Effects of tea consumption on nutrition and health were the focus of investigations over the last decades. Surprisingly, its safety was not paid so much attention. Abd El-Atya et al. reviewed tea contamination with pesticides, heavy metals, polycyclic aromatics, microorganisms, radionuclides, plant growth regulators, and mycotoxins. According to the authors, most contaminants leached into the tea brew are not detected or are found at a level lower than the regulatory limits and do not pose a public health hazard [6]. However, up to date research proved the problem of mycotoxins in tea is worth paying attention to, as shown in Table 1.

Most referenced articles describe the determination of the most toxic mycotoxins, such as aflatoxins and/or ochratoxin A, and fumonisins. Few studies focus on multi-mycotoxin contamination.

Aflatoxins, ochratoxin A, and fumonisins were found in *black tea* samples. Aflatoxin B1 mean concentration achieved 10 µg/kg [30]. The extremely high concentration of ochratoxin A was found in black tea from the Czech Republic—up to 250 µg/kg [39]. Practically all investigated black tea samples from Portugal were contaminated with fumonisins at the level of hundreds of ppb [19]. At the same time, almost “clear” black tea samples were reported in Korea [31]. Our group studied the occurrence of 21 mycotoxins (aflatoxins В1, В2, G1, G2, ochratoxin A, zearalenone, fumonisins B1, B2, T-2 and HT-2 toxins, nivalenol, deoxynivalenol, 3 acetyl- and 15-acetyl deoxynivalenol, diacetoxyscirpenol, fusarenone X, α-и β-zearalenol, citrinin, sterigmatocystin) in 26 black and 4 green loose tea samples. Only two black tea samples were contaminated: sterigmatocystin was detected at near LOD (limit of detection) concentrations [32]. *Green tea* samples were the least contaminated. ELISA examination of four *white* and *red tea* samples from Spain revealed extremely high levels of aflatoxins, ochratoxin A, zearalenone, deoxynivalenol, T-2 toxin, and citrinin [34]. As far as mycotoxins were detected by the method exhibiting the propensity for cross-reactions and overestimation, these results we propose to consider qualitative.

*Pu-erh tea*, due to specific features of its manufacturing, is more contaminated than other kinds of tea. Aflatoxins, ochratoxin A, fumonisins, T-2 toxin, and deoxynivalenol were often found, especially in samples purchased in China. Contamination levels reached dozens and even hundreds of ppb. High levels of deoxynivalenol, fumonisins, and T-2 are doubtful because of the detection method used [36]. Thirty-six samples of Pu-erh tea purchased in the EU proved to be aflatoxin-free, whereas ochratoxin A occurred in 11% of samples, and contamination reached 94.7 µg/kg [28]. Multi-analyte analysis of 31 Pu-erh tea samples by HPLC-MS/MS revealed the presence of a wide spectrum of “unusual” mycotoxins, among them patulin, common for fruit matrixes. It was found in 9 of 15 raw samples (mean concentration is 1169 μg/kg) and in 2 of 16 ripened samples (915 μg/kg) and accounted for *Penicillium citrinum* [26].

The occurrence of ochratoxin A in *bagged tea* was up to 60%: mean ochratoxin A content in the samples from Italy was about 6 µg/kg for 16 samples of black tea and 7 µg/kg for 16 samples of green tea [16]. Pallares with colleagues studied contamination of tea bag infusions with 17 mycotoxins (aflatoxins, ochratoxin А, zearalenone, toxins Т-2 and НТ-2, deoxynivalenol and its acetyl derivates 3-acetyl- and 15-acetyl deoxynivalenol, nivalenol, enniatins A, A1, B, B1, and beauvericin). Raw tea was not tested. Beverages prepared from black and green tea bags were mycotoxin-free, except two green tea samples contaminated with enniatin B at near LOD levels. Aflatoxins В2 and G2 and trace amounts of aflatoxins G1 and 15-acetyl deoxynivalenol were found in brewed green tea with mint [38]. A high aflatoxin B1 concentration level was observed in linden and jasmine herbal tea [40,41].

Monbaliu with colleagues detected 27 mycotoxins (aflatoxins В1, В2, G1, and G2, ochratoxin А, zearalenone, fumonisins В1, В2, В3, toxins Т-2 and НТ-2, deoxynivalenol and its 3- and 15-acetyl derivates, de-epoxy-deoxynivalenol, diacetoxyscirpenol, nivalenol, neosolaniol, fusarenone Х, zearalenone, citrinin, sterigmatocystin, fumigaclavine, mycophenolic acid, paxillin, alternariol, alternariol monomethyl ether, altenuene) in 91 samples of different tea kinds and 76 µg/kg of fumonisin B1 was detected in 1 mix of Ceylon tea only [35].

## 4. Mycotoxin Transfer from Raw Tea into the Beverage

Several parameters define mycotoxin concentration in tea beverages: raw tea contamination level, mycotoxin thermal stability, and its ability to transfer from the matrix into aqueous infusions. Brewing is unable to degrade common mycotoxins [42,43] substantially. There are several water-soluble mycotoxins—aflatoxins (10–20 mg/mL), fumonisins (at least 20 mg/mL), zearalenone (0.02 µg/mL) [44], ochratoxin A (0.0004 mg/mL as acid, water-soluble as salt), T-2 toxin (~0.1 mg/mL), deoxynivalenol (55 mg/mL) [45]—that are able to be extracted from the matrix by water. 

Mycotoxin transfer from naturally contaminated tea into beverage was studied for ochratoxin A only and corresponded to 35–40% in the case of raw black tea [39,46]. Transfer from bagged black and green tea was 34 ± 4% and 54 ± 15% [16]. Fruit tea brewing revealed a 10-fold lower leakage of mycotoxin, which accounted for a lower pH of the resulting infusion and presence of ochratoxin A in molecular form, that is almost water insoluble [39]. Aflatoxin transfer from artificially contaminated samples was found to be 28–33% [47]. Monbaliu et al. failed to detect fumonisin B1 in beverages after brewing of a naturally contaminated raw sample (76 µg/kg) despite high water solubility of fumonisins, and they attributed this fact to low sensitivity of the method [35]. Multi-mycotoxin analysis of tea prepared from tea bags revealed aflatoxins and 15-acetyl deoxynivalenol, as shown in Table 1 [38].

## 5. Exposure Assessment and Legislation 

Aflatoxin B1 and ochratoxin A are the most toxic mycotoxins; their maximum levels in food commodities are set at ppb levels. To evaluate approximate dietary exposure to these contaminants due to tea consumption, we assumed the following: average daily tea consumption—3 cups per day, 2 g of raw tea is used to prepare 1 cup of tea, average body weight is 60 kg, ochratoxin A and aflatoxin average transfer from raw tea into beverage—30%. Average and maximum contamination levels, as shown in Table 2, were estimated based on combined data from several publications using lower bound approach. This exposure assessment is approximate, aimed to draw attention to the problem. Median contamination appeared to be below LOD, that is <0.5 µg/kg for modern methods of analysis. It corresponds to <0.02 ng/kg bw/day—<5% of the average of the lowest dietary exposure from staple food—for aflatoxin B1, and <0.11 ng/kg bw/week—<1%—for ochratoxin A. This impact can be considered as negligible. However, average contamination level results in 3–40% of aflatoxin B1 and 5–24% of ochratoxin A dietary intake compared to the lowest and the highest ends of the range of diet exposure. Compared to ochratoxin A provisional tolerable weekly intake, it corresponds to 0.7–1.7%. Exposure from tea calculated on the base of maximum mycotoxin content exceeds the highest average dietary exposure.

However, mycotoxins in tea are almost not regulated, except for several countries. National regulations concerning mycotoxins in tea have been established in Customs Union countries (Armenia, Belarus, Kazakhstan, Kyrgyzstan, and Russia) for aflatoxin B1 in raw tea—5 µg/kg [50], Argentina set limits for aflatoxin B1 and total aflatoxins in materials used for herbal tea infusions at 5 and 20 µg/kg, correspondingly [51]. Upper limits for such category as “all foods” have been set in Asian countries: in India for aflatoxin B1—30 µg/kg; in Sri Lanka—the same level for total aflatoxins; in Japan—10 µg/kg [52]; and in China—5–20 µg/kg depending on the food matrix [53]. Lack of occurrence data does not allow the conclusion for the necessity of public health protection measures. It is necessary to perform representative studies of different kinds of tea for regulated mycotoxins at least.

## 6. Methods of Mycotoxin Determination in Tea

Several parameters are defining an analytical procedure for mycotoxin determination in tea. First, one should decide whether to analyse raw material or prepared brewing. Permitted levels are set for raw material, while a consumer is much more interested in beverage safety. Recent studies are focused on both matrixes [16,39]. Regardless, the procedure involves sampling, extraction, extract purification, and mycotoxin determination. Raw tea sample preparation is analogous to the standard procedure of mycotoxin determination in food samples. The most common solvents used for extraction are methanol-water and acetonitrile-water, as shown in Table 3, with up to 80% of organics. Additional reagents are sometimes used for extraction, such as NaCl [20] or Tween-20 [47]. The choice of the clean-up method depends on the analysis objective: whether it is multi-determination or targeted analysis of several mycotoxins. In the latter case, immunoaffinity columns (IAC) are preferred due to their high specificity and maximal elimination of matrix components [20,28,30,39,41,47]. Conventional solid phase extraction (SPE) cartridges filled with an anion exchanger, such as amino silica, are appropriate for clean-up of weak acids (ochratoxin A, fumonisins) [19,29]. Liquid-liquid extraction with pH change is proposed for ochratoxin A determination; a combination of extraction and clean-up steps is possible due to a different water/organic phase solubility of molecular and anion forms of the analyte [39]. Comprehensive control of food commodities and requirement of occurrence data accumulation for a wide range of mycotoxins make HPLC coupled to mass spectrometry a method of choice. Sample treatment for multi-target determination should provide appropriate recoveries for compounds of different polarity (e.g., deoxynivalenol, logP = −0.7, enniatin B, logP = 6.5 [45]). At the same time, the matrix effect on electrospray ionisation (ESI) efficiency should be minimised or considered. “Dilute and shoot” procedure outcomes proved to be strongly dependent on the analyte/matrix combination [54]. QuEChERS approach (from Quick, Easy, Cheap, Effective, Rugged and Safe), combining extraction and clean-up, was initially developed for pesticide multi-residue analysis [55] and was adopted for a wide range of analytes and matrices [56]. QuEChERS, “dilute and shoot” with or without additional clean-up in dispersive, and pass through mode SPE were thoroughly validated for mycotoxin and pesticide analysis in green tea supplements [57], and raw tea [58] by HPLC coupled to high-resolution tandem mass spectrometric detection (HRMS/MS). Octadecyl silica gel (ODS) showed good potential as a sorbent for dispersive SPE. No clean-up procedure is also possible. It was used for detection of 30 mycotoxins in coffee samples: MeCN (1% acetic acid) extraction without further purification provided sufficient recovery, and matrix-matched calibration ensured accurate quantification of analytes [59].

Reverse-phase HPLC coupled with specific detectors is a method of choice for mycotoxin analysis. Despite not being “up to date”, FLD provide sufficiently sensitive, selective, and low-cost detection of aflatoxins and ochratoxin A. Multi-mycotoxin analysis supposes applying of MS/MS technology: dozens of mycotoxins can be determined in a single run [32,38]. The current trend is the multi-class detection of food contaminants, such as mycotoxins and pesticides [57,58], or mycotoxins, pesticides, packaging, and process-induced contaminants [60] by HRMS/MS technique. The latter research, being not very successful in mycotoxins′ detection, is also worth paying attention to due to the thorough analysis of parameters affecting method performance. The crucial point of LC-MS with ESI is analyte ionization suppression/enhancement in the presence of matrix components. Tea exhibits the strongest matrix effect, which manifests itself, in a changing fragmentation pattern of mycotoxin ochratoxin A [58]. Matrix effect can be accounted for by such major co-extracts as polyphenols, caffeine, and amino acids [60]. For example, the average caffeine content in tea is about 3.5% [62], that exceeds a minor contaminate concentration (e.g., 10 µg/kg) by over 10^6^ times. Matrix-matched calibrations [38,60] or internal standards [35] are usually used to overcome the problem.

Few studies were focused on tea brew analysis for the reason of mycotoxin transfer estimation [16,39,46] or analytical method development [35,38,60]. Mycotoxin extraction from brewing was carried out by means of IAC (ochratoxin A targeted analysis) [28,39] or dispersive liquid-liquid microextraction (16 mycotoxins, recovery from 66% to 127%) [38] or liquid-liquid extraction and “dilute and shoot” approach (proved to be suitable for ochratoxin A detection) [60]. Hence, the authors followed their preference while preparing tea brew, in short, 1.5–2 g of ground raw tea sample was put in contact with 150–250 mL of boiling water for 3–8 min [16,35,38,39] or Turkish traditional brewing [46]; it is reasonable to propose standard procedure [60,63].

## 7. Conclusions

Camellia sinensis is cultivated and processed in subtropics preferentially. Warm and wet climate also suits mould growth. However, no field fungi were reported in the samples from the local markets in Europe. Storage mycoflora was identified in black and green tea samples. Pu-erh post-fermented tea is characterized by ripening mycoflora. *Aspergillus* and *Penicillium* genera species dominate in both.

Thus, most of the mycotoxin assays were aimed at aflatoxin or ochratoxin A determination. Average aflatoxin B1 concentration raised to 10 μg/kg, ochratoxin A—to 33 μg/kg Modern techniques make possible multi-mycotoxin analysis. Deoxynivalenol and its acetyl derivatives, fumonisins, and enniatin B were found in raw tea samples, supporting the idea of field fungal invasion. Tea beverage safety depends on raw tea contamination level and ability of the mycotoxin to transfer from loose tea into brewing. It was well studied for ochratoxin A only. Compared to raw tea, 30–50% of ochratoxin A was found in the beverage. Aflatoxins and deoxynivalenol leak into brewing also. Extreme toxicity of aflatoxins and occasional occurrence of highly contaminated samples highlight evaluation of their transfer from naturally contaminated samples essential.

Approximate exposure estimates to aflatoxin B1 and ochratoxin A due to tea consumption were carried out based on the combined results of several black and Pu-erh tea surveys. Average contamination level corresponds to the exposure of 3–40% (aflatoxin B1) and 5–24% (ochratoxin A) of mean overall estimates for different cluster diets.

Lack of reliable occurrence data does not allow the conclusion for the necessity of public health protection measures. It is necessary to perform representative studies of different kinds of tea for regulated mycotoxins at least.

## Figures and Tables

**Table 1:** The occurrence of mycotoxins in tea.

Tea	Country	Mycotoxin Positive/All Samples	Mycotoxin Content, μg/kg	LOD/LOQ, µg/kg	Ref.
**Loose or Bricked Tea**					
**Black**	Portugal	FBs: 16/18	80–280 (average *: 149)	20/-	[19]
	Turkey	FBs:5/51	>LOD, <LOQ	31/468 (FВ1), 103/1562 (FВ2)	[29]
	Iran	AFL В1: 11/40 AFL В2: 2/40 AFLG1: 0/40 AFL G2: 3/40 Σ AFL: 11/40	average: 10 average: 12.1	1.0/- (AFL B1, G1), 0.2/- (AFL В2, G2)	[30]
	Korea	AFL: 1/9	1.45	-/-	[31]
	Russia	STC: 2/26 20 МТ: 0/26	0.4; 0.4 n.d.	0.1-50/-	[32]
**Green**	Turkey	FBs: 0/3	n.d.	31/468 (FВ1), 103/1562 (FВ2)	[29]
	Italy	AFL: 0/6	n.d.	0.5/- (AFL B1, G1) 0.2/- (AFL В2, G2)	[20]
	Brazil	AFL: 1/9	<LOQ	-/1	[33]
	Russia	21 МТ: 0/4	n.d.	0.1–50/-	[32]
	Germany	ОТА: 1/32	1.3	-/-	[31]
**Red, White**	Spain	AFL: 4/4 ** ОТА: 4/4 ** ZEN: 4/4 ** Т-2: 4/4 ** DON: 4/4 ** CIT: 3/4 ** FBs: 0/4 **	94.2–853.4 3.7–4.9 4.5–11.2 34.5–42.8 149.1–259.1 18.0–22.3 <LOD	1.4 (ΣAFL) 0.025 (ОТА) 0.14 (ZEN) 0.28 (T-2) 14.8 (DON) 16.5 (CIT) 83 (FBs)	[34]
**White, Yellow, Green, Oolong, Black, Pu-erh, Herbal teas**	Belgium, China	FВ1: 1/91 27 МТ: 0/91	76 n.d.	2–122/- (raw tea), 0.4–46/-μg/L (beverage)	[35]
**Pu-erh**	Austria	AFL В1, FBs:0/36, ОТА: 4/36	n.d. 0.65–94.7 (average: 3.08)	1.7/- (Σ AFL), 10/- (FBs), 0.5/- (ОТА)	[28]
	China	AFL В1: 70/70 FBs: 70/70 ** Т-2: 70/70 ** DON: 70/70 **	0.02–8.5 16–499 5.2–47.7 357–2914	-/- AFL: HPLC-FLD (postcolumn deriv.) FBs, Т-2, DON: ELISA	[36]
	China	AFL В1: 21/30 AFL В2: 4/30 AFLG1: 15/30 AFL G2: 3/30	0.4–15.1 0.1–6.3 0.4–19.0 9.9–56.6	-/- HPLC-FLD (precolumn deriv.)	[37]
**Tea Bags**					
**Black**	Italy	ОТА: 11/16	1.4–21.5 (average: 6.3)	0.01	[16]
	Spain ***	17 МТ: 0/12	n.d.	LOD: 0.05–10 μg/L LOQ: 0.2–33 μg/L	[38]
	Czech Republic	ОТА: 4/12	1.9–250 (average: 33.1)	LOD: 0.1, LOQ: 0.35	[39]
**Green**	Italy	ОТА: 14/16	0.1–20.0 (average: 7.2)	LOD: 0.01	[16]
	Spain ***	ENN В: 2/10 16 МТ: 0/10	~LOQ (~0.2 µg/L) n.d.	LOD: 0.05–10 μg/L LOQ: 0.2–33 μg/L	[38]
**Green + Mint**		AFL В2: 6/8 AFL G1: 4/8 AFL G2: 4/8 15-acetyl DON: 2/8 13 МТ: 0/8	14.4–32.2 (average: 26) ~LOQ (~2.4 µg/L) 1.9–2.6 (average: 2.3) 60.5; 61 n.d.		
**Red**		17 МТ: 0/14	n.d.		

Notes: MT—mycotoxins, AFL—aflatoxin, FBs—fumonisins, FВ1—fumonisin В1, STC—sterigmatocystin, ОТА—ochratoxin А, DON—deoxynivalenol, ENN В—enniatin В, Т-2—Т-2 toxin, CIT—citrinin, ZEN—zearalenone; LOD—limit of detection, LOQ—limit of quantification, n.d.—not detected. * an average of all tested samples. ** sample contamination was examined by ELISA, the results are rather qualitative, than quantitative. *** mycotoxins were detected in brewed tea.

**Table 2:** Exposure estimate to aflatoxin B1 and ochratoxin A from tea.

Mycotoxin	Tea	Data Numberof Samples, Origin	Contamination *, μg/kg			Exposure, ng/kg bw				Dietary Intake, ng/kg bw
			Median	Mean	Max		Median	Mean	Max	
**Aflatoxin B1**	Black	40, Iran [30] 9, Korea [31] 26, Russia [32]	<LOQ	5.3	190	**per day**	negligible	0.16	5.7	0.4–2.6 [48]
	Pu-erh	36, Austria [28] 30, China [37] 70, China [36]	1.6	2.6	15.1		0.05	0.08	0.45	
**Ochratoxin A**	Black	12, Czech Republic [39] 26, Russia [32] 16 (bagged), Italy [16]	<LOQ	9.2	250	**per week**	negligible	1.93	52.5	8–17 [49]
	Pu-erh	36, Austria [28]	<LOQ	3.8	94.7		negligible	0.80	19.9	

* Lower bound approach was used to calculate means, i.e., samples below LOD or LOQ were considered as zero.

**Table 3:** Methods of mycotoxin determination in tea.

Detection (Mycotoxin)	Tea	Publication. Year	Short Description		Sensitivity, μg/kg **	Ref.
			Extraction/Clean up	HPLC/Detection		
**TLC-FLD (AFL, ОТА, ZEN)**	Herbal raw	1998	MeCN:Н_2_О (9:1, *v*/*v*), CHCl_3_ alkalinization/acidification	TLC: silica gel toluene, ethyl acetate, formic acid (50 + 40 + 10 *v*/*v*). UV irradiation	-	[17]
**HPLC-FLD (FBs)**	Black raw	2001	MeOH:Н_2_О (3:1, *v*/*v*), SPE: anion-exchange column	Derivatisation (o-phthaldialdehyde), HPLC: ODS FLD, λ_Ex/Em_ = 335 /440 nm	31/468 (FВ1), 103/1562 (FВ2)	[19]
**HPLC-FLD (FBs)**	Black, green raw	2004	MeOH:Н_2_О (3:1, *v*/*v*), SPE: anion-exchange column	Derivatisation (o-phthaldialdehyde), HPLC: ODS FLD, λ_Ex/Em_ = 338/455 нм	LOD: ~30 (FВ1), ~470 (FВ2)	[29]
**HPLC (AFL)**	Herbal, green raw	2007	MeOH:Н_2_О (80:20, *v*/*v*), NaCl, SPE: IAC	HPLC: ODS Post-column derivatisation (Br_2_, Cobra cell) FLD, λ_Ex/Em_ = 365/435 nm	LOD: 0.5 (AFL B1, G1) 0.2 (AFL В2, G2)	[20]
**ELISA (ОТА, FBs, ΣAFL, ZEN, Т-2, DON, CIT)**	Red, white raw	2009	*AFL, ZEN, Т-2, DON:* MeCN:Н_2_О (84:16, *v*/*v*), SPE: IAC (AFL + ZEN, DON + Т-2) *FBs:* MeCN:Н_2_О (50:50, *v*/*v*), SPE: IAC *CIT*: H_3_PO_4_:CH_2_Cl_2_, SPE: polyamide column *ОТА:* CH_2_Cl_2_ alkalinisation/acidification	ELISA λ = 450 nm	LOD: 0.025 (ОТА) 83 (FBs) 1.4 (ΣAFL) 0.14 (ZEN) 0.28 (T-2) 14.8 (DON) 16.5 (CIT)	[34]
**UHPLC-MS/MS (27 Mycotoxins)**	White, green, yellow, black, oolong, Pu-erh raw and beverage	2010	Ethyl acetate: formic acid (99:1, *v*/*v*), SPE: amino-и ODS	UHPLC: ODS MS/MS: electrospray, positive polarity, internal standards	LOD: 2–122 (raw tea), 0.4–46 μg/L (beverage)	[35]
**HPLC (AFL)**	Black raw	2012	MeCN:MeOH:Н_2_О (10:6:4, *v*/*v*), 8% Tween-20. SPE: IAC	HPLC: ODS Post-column photochemical derivatization, FLD, λ_Ex/Em_ = 360/440 nm	LOD: 0.3 μg/L (AFL B1, G1) 0.15 μg/L (AFL В2, G2)	[47]
**HPLC-FLD (AFL)**	Green raw	2012	Acetone:Н_2_О (85:15) SPE: IAC	HPLC: ODS Post-column derivatisation (Br2, Cobra cell) FLD, λ_Ex/Em_ = 360/435 nm	LOQ: 1	[33]
**ELISA, HPLC, HPLC-MS (AFL, ZEN, OTA)**	Pu-erh raw	2013	AFL:MeOH:Н_2_О (70:30, *v*/*v*) + Tween-20, SPE: IAC FBs: MeCN:MeOH:Н_2_О (1:1:2 *v*/*v*), SPE: IAC ОТА: MeСN:Н_2_О (80:20, *v*/*v*), SPE: IAC	AFL: HPLC: ODS Post-column derivatization (Br2, Cobra cell) FLD, λ_Ex/Em_ = 362/440 nm FBs: HPLC: ODS, MS/MS ОТА: HPLC: ODS FLD, λ_Ex/Em_ = 333/460 nm	LOD: 1.7 (Σ AFL), 10 (FBs), 0.5 (ОТА)	[28]
**ELISA (AFL)**	Herbal raw	2013	MeOH:Н_2_О (70:30, *v*/*v*)	ELISA	LOD: 1.7 (Σ AFL), 1.0 (AFL B1)	[40]
**HPLC-FLD (AFL)**	Black raw	2013	MeOH:Н_2_О (80:20, *v*/*v*) + NaCl, SPE: IAC	HPLC: ODS Post-column derivatization (Br2, Cobra cell) FLD, λ_Ex/Em_ = 360/435 nm	LOD: 1.0 (AFL B1, G1), 0.2 (AFL В2, G2)	[30]
**HPLC-FLD (ОТА)**	Black raw, beverage	2014	Raw: CHCl_3_ + alkalinisation/acidification SPE: IAC Beverages: SPE: phenyl silica gel	HPLC: ODS FLD, λEx/Em = 333/465 nm	LOD: 0.1 LOQ: 0.35	[39]
**HPLC (AFL В1)ELISA (FВ1, DON, Т-2)**	Pu-erh raw	2014	MeOH:Н_2_О (70:30, *v*/*v*), SPE: IAC (AFL B1)	FВ1, DON, Т-2: ELISA AFL В1: HPLC: ODS Post-column derivatisation (iodine), FLD	-	[36]
**HPLC, ELISA (AFL)**	Pu-erh raw	2015	MeCN:Н_2_О (84:16, *v*/*v*), SPE	ELISA HPLC: ODS Precolumn derivatisation (TFA) FLD, λ_Ex/Em_ = 360/440 nm	- calibration curve lowest level corresponds to 1	[37]
**HPLC-HRMS/MS (55 Mycotoxins)**	Raw tea	2015	QuEChERS dispersive SPE: ODS	HPLC: ODS HRMS/MS, electrospray	1–1000	[58]
**HPLC-FLD (AFL)**	Herbal raw	2016	MeOH:Н_2_О (6:4, *v*/*v*) with NaCl, SPE: IAC	HPLC: ODS Post-column derivatisation (Br_2_, Cobra cell analogue) FLD, λ_Ex/Em_ = 362/440 nm	LOD: 0.1 (AFL B1, G1) 0.02 (AFL В2, G2)	[41]
**HPLC-MS/MS, (16 Mycotoxins)**	Black, green, red, green + mint beverage	2017	Dispersive liquid-liquid microextraction by MeCN-ethyl acetate and MеОН-chloroform	HPLC: ODS MS/MS: electrospray, positive polarity, matrix match calibration	LOD: 0.05–10 μg/L LOQ: 0.2–33 μg/L	[38]
**HPLC-MS/MS, (22 Mycotoxins)**	Black, green	2018	MeCN: Н_2_О:acetic acid (79:20:1)	HPLC: ODS MS/MS: ESI, positive polarity	LOD: 0.1–50	[32]
**HPLC-FLD (ОТА)**	Tea bags	2018	MeOH/NaHCO_3_aq. 1%, (70/30) SPE: IAC	HPLC: ODS FLD, λ_Ex/Em_ = 333/466 nm Precolumn derivatisation (BF_3_) for positive findings confirmation Internal standard: diflunisal	LOD: 0.01	[16]
**HPLC-HRMS/MS (4 Mycotoxins Tested)**	Green tea raw, brew	2018	-QuEChERS without dSPE (raw) -MeCN, Н_2_О, MgSO_4_, NaCit, 5-fold dilution prior analysis (brew) -MeCN, Н_2_О, formic acid (brew)	HPLC: ODS HRMS/MS, electrospray	FBs—n.d. LOQ (DON) = 500 LOQ (OTA) = 10	[60]

* TLC—thin layer chromatography, SPE—solid-phase extraction, dSPE—dispersive SPE, IAC—immunoaffinity column, ODS—octadecyl silica gel, FLD—fluorimetric detection, λ_Ex/Em_—excitation/emission wavelength, TFA—trifluoroacetic acid, ESI—electrospray ionisation, HRMS/MS—high-resolution tandem mass spectrometric detection. **—for HPLC-MS/MS method validation most of the authors follow SANTE requirements since 2015: SANTE 11945/2015 and its later upgrade SANTE/11813/2017 [61].
